# The Role of Palliative Surgery for Malignant Bowel Obstruction and Perforation in Advanced Microsatellite Instability-High Colorectal Carcinoma in the Era of Immunotherapy: Case Report

**DOI:** 10.3389/fonc.2020.00581

**Published:** 2020-04-21

**Authors:** Sean J. Judge, Jingran Ji, James Liu, Manmeet Kaur, Edward Kim, Jun Gong, Kit W. Tam, Amanda R. Kirane, Sepideh Gholami, Robert J. Canter, Richard J. Bold, Alexandra Gangi, Marwan Fakih, May Cho

**Affiliations:** ^1^Division of Surgical Oncology, Department of Surgery, University of California, Davis, Sacramento, CA, United States; ^2^Department of Internal Medicine, University of California, Davis, Sacramento, CA, United States; ^3^Division of Hematology and Oncology, Department of Medicine, University of California, Davis, Sacramento, CA, United States; ^4^Division of Hematology and Oncology, Department of Medicine, Cedars-Sinai Medical Center, Los Angeles, CA, United States; ^5^Division of Surgical Oncology, Department of Surgery, Cedars-Sinai Medical Center, Los Angeles, CA, United States; ^6^Division of Hematology and Oncology, Department of Medicine, City of Hope National Medical Center, Duarte, CA, United States

**Keywords:** palliative surgery, metastatic colorectal adenocarcinoma, microsatellite instability high (MSI-H), immunotherapy, malignant bowel obstruction

## Abstract

The role of palliative surgery in the management of acute complications in patients with disseminated malignancy remains controversial given the complexity of assessing acute surgical risk and long-term oncologic outcome. With the emergence of checkpoint blockade immunotherapy, there appears to be an increasing role for historically palliative procedures as a bridge to systemic immunotherapy. This is especially evident in advanced microsatellite instability-high (MSI-H) colorectal cancer where malignant obstruction and fistula formation are more common and where immunotherapy with checkpoint blockade (anti-PD-1/PD-L1, anti-CTLA-4) has a high response rate with potential for favorable oncologic outcomes. We present a series of three patients with MSI-H metastatic colorectal cancer complicated by malignant bowel obstruction and fistula formation, who having progressed on standard chemotherapy, underwent palliative intervention as a bridge to immune checkpoint blockade with durable and clinically meaningful anti-cancer responses. These cases highlight the need to re-evaluate the role of historically palliative operations in the setting of disease progression for immunotherapy-responsive tumors.

## Introduction

Surgery as a component of palliative care in the setting of metastatic colorectal cancer (mCRC) has been, and remains, controversial due to a narrow risk/benefit ratio with the potential for significant postoperative morbidity and mortality impacting quality of life, performance status, and tolerability of additional anti-cancer therapies (i.e., cytotoxic chemotherapy). The decision to intervene surgically for patients with disseminated malignancy continues to revolve around various challenging medical and ethical considerations including disease burden, trajectory of disease progression with and without systemic therapy, availability of efficacious systemic treatment options, and patients' goals of care (1). Our previous work analyzing a large statewide database indicated that compared to medical management, surgical management of malignant bowel obstructions was associated with increased complications, increased in-hospital mortality, and lower rates of discharges to home (2). This is made more difficult by the finding that common risk stratification tools (American Society of Anesthesiology classification, Charlson comorbidity index, and modified frailty score), do not adequately predict postoperative adverse events in this population (3). These considerations are especially challenging for patients receiving chemotherapy, as both invasive surgery and chemotherapy require fit patients with good performance status to optimize the risk/benefit ratio of multimodality therapy.

One subset of colorectal cancer patients where this risk/benefit ratio is changing is in those with tumors exhibiting microsatellite instability-high (MSI-H) genotypes—a marker of dysfunctional DNA repair proteins, which can represent either somatic or germline (i.e., Lynch Syndrome) mutations. Given the high rate of response and durability of response with immune checkpoint blockade in MSI-H (also known as mismatch repair deficient (dMMR) when determined by IHC) colorectal cancer patients (where a high tumor mutational burden leads to partial and complete responses in ~30–50% of patients), palliative interventions may alleviate acute surgical processes, and rather than strictly improving quality of live, can act as a bridge to immunotherapy with the potential for prolonged survival.

The most common palliative surgical intervention in advanced colorectal cancer remains stoma creation for relieving malignant bowel obstruction and its associated symptoms. Ostomy creation does not provide a cancer-related survival benefit, but does provide symptom relief and can improve quality of life (4). The role of other invasive surgical procedures in the setting of malignant bowel obstruction remains controversial. A Cochrane review investigating the efficacy of surgery for bowel obstruction in advanced cancers was published in 2016 (5). This analysis of 43 studies, including 4,265 patients, found a wide distribution of results, with 26.7 to 68% of patients obtaining clinical resolution of symptoms after surgery. More so, rates of re-obstruction ranged from 0 to 63%, but data on the time to re-obstruction was limited. The authors of this large analysis concluded that the data are too limited to make any conclusions on surgical management recommendations (5). A separate analysis evaluating the literature on type of palliative surgery for obstruction in patients with peritoneal carcinomatosis found an overall survival (OS) of 6.4 months after palliative surgery. On subset analysis, patients who underwent tumor resection had an OS of 7.2 months and those that underwent enteral bypass had an OS of 2.7 months (6). These analyses included studies evaluating a heterogenous cohort of colorectal cancer patients, which included both microsatellite stable (MSS) and MSI-H tumor types. These studies also preceded the era of immunotherapy where patients with MSI-H/MMR deficient pathogenesis can have significant responses to checkpoint blockade therapy. Given these findings, there is likely greater utility for palliative surgery in MSI-H colorectal cancer patients given the potential for durable responses and prolonged survival benefits with immunotherapy.

MSI-H tumors represent about 5% of mCRC cases and are associated with a poor prognosis in comparison to MSS tumors (7, 8). These tumors tend to invade deeper and are poorly differentiated on histology (9, 10). In the pre-immunotherapy era, recurrent MSI-H disease also carried worse OS from diagnosis to death (HR: 1.363, *P* = 0.035) as well as OS from recurrence to death (HR: 2.667, *P* < 0.001) compared to MSS disease (11, 12). Profound and durable responses with immunotherapy, however, has changed the expected OS of MSI-H CRC patients. Le et al. demonstrated in a phase II study that MSI-H/dMMR mCRC patients who received pembrolizumab (anti-PD-1) achieved a 90% disease control rate, 78% immune-related progression-free survival (PFS) at 20 weeks, and an objective response rate of 30–40% (13). Nivolumab (anti-PD-1) has also shown clinical benefit [overall response rate (ORR), 31%; disease control rate, 69%; 12 month OS, 73%] in previously treated patients with MSI-H/dMMR mCRC (14). Dual agent checkpoint blockade therapy also appears promising in MSI-H/dMMR disease as a recent cohort analysis from the CheckMate-142 trial who were treated with nivolumab/ipilimumab (anti-PD-1 with anti-CTLA-4) showed an ORR of 55%, 12 week disease control rate of 80%, PFS rate of 71% and OS of 85% after 1 year (15). Notably, there was a 3% complete response (CR) rate with combination therapy.

While reported response rates are promising, some critical factors limit the efficacy of this treatment. Currently, checkpoint blockade therapy is not an approved first-line agent in mCRC, therefore, patients presenting with acute complications after progressing on cytotoxic therapy require more durable interventions if they are to start immunotherapy. Additionally, unlike traditional cytotoxic therapies, time to response to immunotherapy can be longer. In recent clinical trials, for example, the median time to response for both nivolumab and nivolumab/ipilimumab was 2.8 months, and was 4.8 months for pembrolizumab (13, 14). This creates the scenario where patients must first fail standard chemotherapy before starting immunotherapy, and once on immunotherapy, a clinical benefit may not be determined for weeks to months. The challenge in this patient population is that without a more durable surgical intervention for acute complications of mCRC, MSI-H patients may miss the opportunity to start and maintain potentially life-prolonging therapies. Given the dramatic clinical improvements with novel immunotherapeutics, surgical intervention for complications secondary to MSI-H CRC should be considered as a bridge to immunotherapy in select patients, and not strictly for the relief of obstructive symptoms.

**Figure 1:**
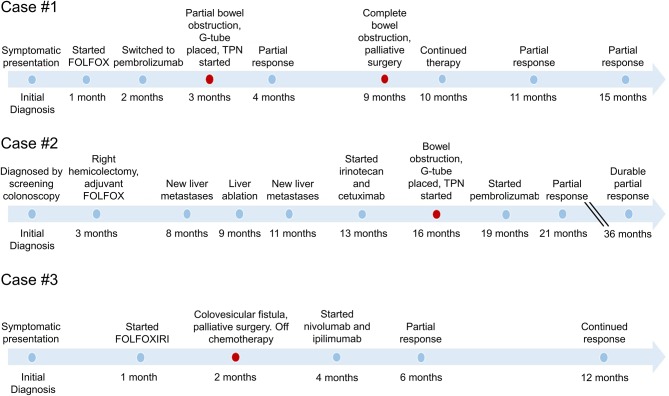
Timeline of events in the care of each patient with MSI-H metastatic colorectal cancer. Timeline demonstrates the variable response to therapy before and after palliative surgery. Palliative surgical interventions are indicated in red, demonstrating the relationship to initiation of immunotherapy and the sustained duration of response.

To this end we present three patients with MSI-H mCRC who developed bowel obstructions or fistulae related to their malignancy while on chemotherapy. After significant deliberation with each patient and multidisciplinary treatment planning, two patients underwent palliative surgical interventions and one underwent an interventional radiology-based procedure with the intention of acting as a bridge to immunotherapy. These interventions allowed patients to receive and benefit from immunotherapy with durable disease control. Pertinent patient demographic and medical history details are provided in Supplementary Table 1 and the timeline of events is shown in Figure 1.

## Case Description

### Case 1

**Figure 2:**
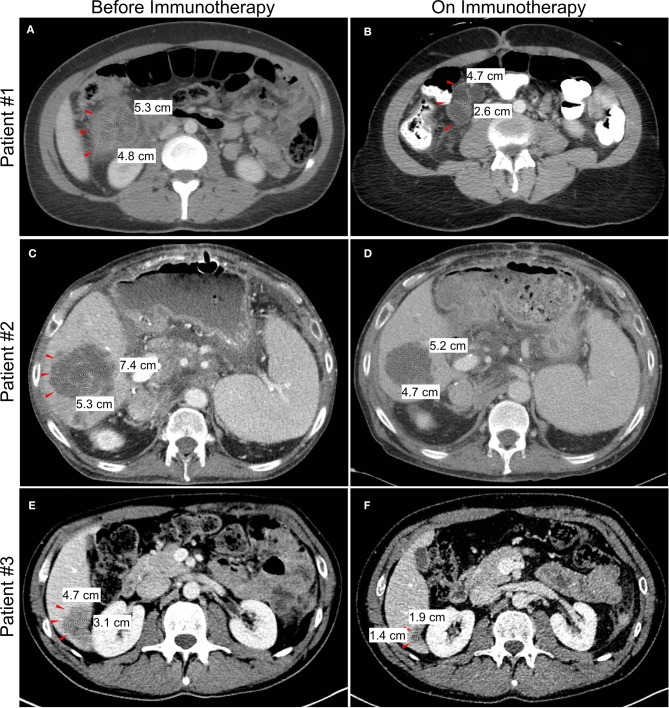
Changes in tumor or metastasis size by computed tomography (CT) imaging before and after initiation of checkpoint blockade immunotherapy. Representative imaging showing decrease in size of colon mass for patient #1 **(A,B)** and liver metastasis for patient #2 **(C,D)** and patient #3 **(E,F)**.

A 33 year-old woman presented with abdominal pain in 2018. Computed tomography (CT) imaging revealed a 3.9 × 3.7 cm heterogenous soft tissue mass in the ascending colon near the hepatic flexure with invasion of the right anterior pararenal fascia and the second/third portion of the duodenum. There were associated enlarged and necrotic portacaval and mesenteric lymph nodes and a 1 cm hypodense lesion in the right hepatic lobe. Colonoscopy with biopsy confirmed a diagnosis of adenocarcinoma, and subsequent immunohistochemistry (IHC) showed loss of MLH1 and PMS2 gene expression consistent with MSI-H/dMMR. The patient was started on 5-fluorouracil, leucovorin, and oxaliplatin (FOLFOX), but continued experiencing symptoms of partial bowel obstruction including severe nausea, emesis, abdominal pain and dizziness from hypovolemia. CT imaging at this time is shown in Figure 2A. Given no evidence of response to chemotherapy for 2 months, she was transitioned to pembrolizumab (anti-PD-1). Shortly after initiation of pembrolizumab, she developed a partial large bowel obstruction secondary to the tumor. This was relieved by placement of a percutaneous gastrostomy tube and she was started on total parenteral nutrition (TPN) and resumed immunotherapy. Following three cycles of immunotherapy, imaging was consistent with partial response in the primary tumor and associated lymph nodes and the patient was also able to transition to an oral diet. However, after 8 months on immunotherapy she developed complete bowel obstruction, though imaging showed continued response in the primary tumor and metastases. The bowel obstruction was thought to be from adhesions and inflammatory changes and not progression of the patient's primary disease. Following multidisciplinary discussions between the patient and her treatment team including Medical Oncology, Surgical Oncology, and Palliative Care, the decision was made to proceed with surgical intervention. She underwent an internal colonic bypass with anastomosis of the cecum to the transverse colon bypassing the inflamed segment. She recovered without complications and was able to resume oral alimentation with resumption of pembrolizumab monotherapy. At the time of this report, 14 months since initiating pembrolizumab, imaging demonstrates continued partial response on immunotherapy (Figure 2B).

### Case 2

A 62 year-old man with a history of liver transplantation for alcoholic cirrhosis underwent screening colonoscopy and was found to have moderately differentiated adenocarcinoma of the ascending colon. Molecular analysis identified loss of MLH1 and PMS2 genes, consistent with MSI-H/dMMR disease. The patient initially underwent a right hemicolectomy, and surgical pathology revealed AJCC stage IIIc (pT3N2b) disease with 7 of 27 lymph nodes positive for adenocarcinoma. He was treated with adjuvant FOLFOX, and in the following 6 months developed hepatic metastases along with diffuse omental and mesenteric disease consistent with failure of first-line therapy (Figure 2C). Additional chemotherapeutic regimens did not produce any response, and he subsequently developed a complete small bowel obstruction and acutely worsening cachexia.

In the setting of concurrent liver dysfunction, prior liver transplantation, and new extensive liver metastases, discussions regarding surgical intervention vs. a less invasive percutaneous option were debated. With the potential to receive immunotherapy and given the increased risk associated with this unique patient, an interventional radiology-guided percutaneous gastrostomy tube was placed to palliate his obstructive symptoms, and he initiated TPN for caloric support. After extensive discussions regarding the unique risks of immunotherapy in the setting of liver transplantation, the patient was started on pembrolizumab. On-treatment follow-up imaging showed a partial response of both the primary and metastatic lesions (Figure 2D) and the patient experienced clinical improvement in his obstructive symptoms after 2 months of therapy. He resumed oral intake with cessation of TPN and continues to receive benefit on pembrolizumab several months later, without any subsequent obstructive episodes. While the risk of undergoing a palliative surgery was felt to be too high in this specific patient, this case highlights the unique risk/benefit profile that must be evaluated in this increasingly complex patient population.

### Case 3

The final case is a 37 year-old man who initially experienced persistent alternating diarrhea and constipation. Within 5 months, these symptoms progressed to urinary retention, and imaging revealed a 5.5 cm sigmoid mass with invasion into the bladder and metastatic liver lesions (Figure 2E). Image-guided percutaneous liver biopsy confirmed metastatic adenocarcinoma from a colon primary. Molecular analysis confirmed MSI-H/dMMR disease, and he was started on 5-fluorouracil, oxaliplatin, leucovorin, and irinotecan (FOLFOXIRI) chemotherapy. Within 2 months of starting chemotherapy he developed a colovesicular fistula, thus limiting his ability to receive and tolerate further chemotherapy. Multidisciplinary discussions were held between colorectal surgery, urology and medical oncology, and it was determined that resection would offer the best chance for managing the fistula, controlling ongoing infections, and resuming cancer-directed therapy. He subsequently underwent an en bloc resection of the sigmoid colon and dome of the urinary bladder with formation of an end colostomy. Following an uncomplicated postoperative recovery, he was started on dual checkpoint blockade, nivolumab (anti-PD-1) and ipilimumab (anti-CTLA-4), with a partial response of the liver metastases (Figure 2F). The patient has continued to benefit from durable response from immunotherapy and has since returned to work.

## Discussion

The three cases presented here highlight the changing landscape of oncology care in the era of immunotherapy for colorectal cancer patients with MSI-H disease and a reasonable expectation of response to immune checkpoint blockade. For those who respond to treatment, advanced MSI-H disease now carries a much different prognosis compared to the pre-checkpoint blockade era. This is dramatically changing the decision-making and management of medical and surgical specialists who provide care to these advanced cancer patients.

In colorectal cancer, checkpoint inhibitors such as pembrolizumab, nivolumab, and ipilimumab have been approved for MSI-H/dMMR metastatic colorectal cancer that has failed standard therapies (13–17). While studies on the benefits of palliative surgical interventions in the setting of malignant obstruction and fistula formation may have only suggested modest improvement in overall survival at best, these interventions were often followed by conventional chemotherapy or best supportive care in non-selected patients. With immune checkpoint inhibitors there appears to be a more significant survival benefit from palliative interventions, including surgery, as a bridge to these specific therapies. The response rates are variable but many patients, such as the ones described here, have had significant and lasting responses. Furthermore, it does not appear that age had a significant impact on response as the trials included patients aged 20–80 years.

Since checkpoint blockade immunotherapeutics are not yet approved for first-line therapy, there is often a significant period on traditional chemotherapy before starting immunotherapy. In the CheckMate-142 trial, 54% of patients had received 3 or more chemotherapy regimens before starting immunotherapy on trial (14). Given the aggressive disease in MSI-H colorectal cancer in addition to its well-known resistance to chemotherapy, patients may present with acute complications or develop bowel obstruction and fistulae during chemotherapy. Another consideration when evaluating these patients for surgical procedures is the adverse effects of therapy and the impact of immunotherapeutics on the safety of surgery. Regarding adverse events, immune-related adverse events (IRAEs) are common with anti-PD-1 and anti-CTLA-4 therapy and patients should be monitored for after initiation of therapy. Common IRAEs include, but are not limited to, colitis, enteritis, pneumonitis and hepatitis. The risk of IRAEs should be determined in a patient-specific manner, and increased risk should not preclude initiation of checkpoint blockade therapy (18). As for surgical complications, a smaller, retrospective study from the Mayo Clinic, Florida was recently published with results of their experience to help answer this question (19). Elias et al. identified 17 patients who underwent 22 unique operations while on perioperative immunotherapy. Although their patient cohort did not include any patients with colorectal malignancies, the authors report five bowel operations with seven bowel anastomoses and no post-operative anastomotic leaks. Elias et al. along with another study examining the safety of perioperative ipilimumab in metastatic melanoma (20), suggest that perioperative immunotherapeutics do not present a distinct contraindication to surgery when indicated.

Our recent experience suggests that interventions that are historically viewed as “palliative” should be re-evaluated and thoughtful consideration be given for surgical interventions as a bridge to immunotherapy in select metastatic colorectal cancer patients with MSI-H disease. Because MSI-H disease is frequently less responsive to standard chemotherapy, intervening for complications such as bowel obstruction and fistulae formation may be more appropriate as this allows for palliation of symptoms and for patients to transition to checkpoint blockade therapy, where response rates are more pronounced. Our experience described in this study is limited by a small sample size and has potential for selection bias. Therefore, the criteria for patient selection, route and timing of intervention requires investigation and should be discussed in a multidisciplinary setting. Further investigation is warranted in this subset of MSI-H mCRC patients to validate the role of palliative interventions that may provide a window for immunotherapy, potentially leading to prolonged and durable responses in this unique patient population.

## Ethics Statement

Written informed consent was obtained from the patients for the publication of any potentially identifiable images or data included in this article.

## Author Contributions

SJ: manuscript, planning, writing, and editing. JJ: case write-up, figure creation, references, manuscript editing, and submission. RC: case submission, surgical expertise, and manuscript editing. JL and MK: manuscript editing and adding references. EK and KT: patient care/submission and manuscript editing. JG and AG: expert opinion, references, and manuscript editing. AK, SG, and RB: surgical expertise, patient care/submission, and manuscript editing. MF: expert opinion and manuscript editing. MC: principle investigator.

## Conflict of Interest

The authors declare that the research was conducted in the absence of any commercial or financial relationships that could be construed as a potential conflict of interest.
