# Supplementary material for: The Role of Palliative Surgery for Malignant Bowel Obstruction and Perforation in Advanced Microsatellite Instability-High Colorectal Carcinoma in the Era of Immunotherapy: Case Report
Source: Front Oncol. 2020 Apr 21;10:581. doi: 10.3389/fonc.2020.00581 (PMC7186327; doi:10.3389/fonc.2020.00581)
Supplement: Supplementary file 1 [file Table_1.pdf]

Supplemental Table 1. Patient demographics and medical history

**Patient**	**Age (years)**	**Gender**	**Diagnosis**	**Chemotherapy Regimen**	**Complication(s)**	**Past Medical and Surgical History**	**Familial Cancer Syndrome?**
1	33	Female	mCRC	FOLFOX	Partial SBO/LBO	None	No
2	62	Male	mCRC	FOLFOX, Irinotecan, Cetuximab	Complete SBO	Alcoholic cirrhosis, orthotopic liver transplant	No
3	37	Male	mCRC	FOLFOXIRI	Colovesicular fistula	None	No

mCRC, metastatic colorectal cancer; SBO, small bowel obstruction; LBO, large bowel obstruction
